# Moving beyond superficial communication to collaborative communication: learning processes and outcomes of interprofessional education in actual medical settings

**DOI:** 10.20407/fmj.2019-026

**Published:** 2020-07-14

**Authors:** Mihoko Ito, Takeshi Hida, Kazue Goto, Michiko Goto, Yoshikiyo Kanada, Masatsugu Ohtsuki

**Affiliations:** 1 Faculty of Rehabilitation, Fujita Health University, School of Health Sciences, Toyoake, Aichi, Japan; 2 Department of Nursing, Ichinomiya Kenshin College of Nursing, Ichinomiya, Aichi, Japan; 3 Center for Medical Education, Fujita Health University, School of Medicine, Toyoake, Aichi, Japan; 4 Department of Family Medicine, Mie University Graduate School of Medicine, Tsu, Mie, Japan; 5 Faculty of Medicine, Fujita Health University, School of Medicine, Toyoake, Aichi, Japan

**Keywords:** Collaborative medical practice, Interprofessional education, SCAT, Interpretive analysis, Communication

## Abstract

**Objectives:**

The current study sought to understand the learning outcomes experienced by students and to explain their learning process in detail using interpretive data analysis.

**Methods:**

A qualitative study examined students who participated in a multidisciplinary course in a ward. This study investigated latent meanings rather than factual information, using an interpretive paradigm. Data were collected via focus groups and analyzed using Steps for Coding and Theorization (SCAT).

**Results:**

Students in the Assembly IV trial (interprofessional education in actual medical settings) experienced a process of transition from a competing (exclusive) mode to a mutual-understanding mode when communicating with people in other professions, and they acquired the perspective of an interactive (dialectic) link between involved communication (communication that attempts to connect directly with patients) and uninvolved communication (communication with patients indirectly through data and other methods) for patient communication. This enabled students to move beyond superficial communication while deepening their connections with people in other professions, complementing each other’s strengths, and learning about the possibilities inherent in the provision of collaborative medical practice.

**Conclusions:**

Students participating in interprofessional education within medical settings learned about the potential to achieve a circular realization of collaborative medical practice. A circular realization of collaborative medical practice involves incorporating diverse approaches into one’s own professional work via exposure to the viewpoints of other occupations and avoiding decision-making based on assumptions that are only valid within one’s own profession. This process enables the discovery of better methods and perspectives and the achievement of effective medical practice by moving beyond superficial communication.

## Introduction

The medical field in Japan is currently experiencing rapid advancement and specialization, and the level of quality and safety demanded by citizens for medical treatment is gradually increasing. Interprofessional work (IPW) has become increasingly important for addressing the complex problems faced by local communities regarding the growing population of older people in Japan’s rapidly aging society. Consequently, there is a need to conduct interprofessional education^1^ (IPE) to prepare medical students prior to graduation. The World Health Organization published a framework for IPE and multidisciplinary cooperation^2^ and is promoting IPE internationally. Recent surveys in Japan have indicated that an increasing number of schools and departments are implementing IPE in the health, medical, and welfare fields.^3^ Teaching methods often consist of small-group study in lectures and classrooms,^3,4^ with few instances of IPE in medical settings.^5,6^ Outcome-based education^7^ focuses on the intended learning outcome at the time of graduation.^8–10^ Various learning outcomes for IPE have been reported,^5,11^ with the core domains for Japan’s IPE framework^12^ being “Patient-/Client-/Family-/Community-centered” and “Interprofessional Communication.”

Since its founding in 1964, Fujita Health University has conducted interdepartmental The Assembly Education for all first- and second-year students to provide a foundation for team-based medical practice. The Assembly Education for first-year students (Assembly I) and second-year students (Assembly II) offers students the choice of a variety of study projects while fostering communication with others. In 2013, team-based learning (TBL) was introduced as a form of IPE for students in their third year or beyond, and The Assembly Education began to also be provided for upper-class students.^13^ The Assembly Education mainly for students in their third year (Assembly III), emphasizing a patient-centric viewpoint and promoting discussion using mock cases. The Assembly Education for first- through third-year students (who have not yet experienced hands-on training in a medical setting) provides participants with opportunities to communicate with their fellow students and instructors, but it does not provide opportunities for learning how to communicate with patients or members of other professions. Therefore, the university began offering Assembly IV on a trial basis in 201x as an elective program for students in their fourth year or beyond who had completed their clinical training for the courses in each faculty.

From September 11 through September 15, 201x, a total of 20 individuals participated in the Assembly IV trial, including two fourth-year students from the school of medicine and 18 fourth-year students from six faculties in the school of health sciences. Participants were divided into three cross-departmental teams and assigned to the university’s hospital wards (nephrology, and emergency and critical medicine) or a nursing home, where they received hands-on training (Table 1). The goal of the training was to give the students an understanding of the roles of different professions so that they could learn about the links between those professions and their own. Students in Group 1 practiced in the nephrology ward. Students in Group 2 practiced at the emergency and critical medicine ward. The students both in Group 1 and Group 2 collected medical information about their assigned patient from the medical records and the staff at the wards, then visited the patient’s room. Students accompanied the in-patient when they underwent examinations (X-rays and electrocardiogram), and observed the actions of staff at the wards in response to changes in the condition of their patient. In addition, students attended a Multidisciplinary Discharge Conference. Students in Group 3 practiced at a nursing home and were put in charge of patients with dementia. They observed the patient’s daily life and gathered information by talking with the patient. Because no medical technologists or radiological technologists were stationed at the facility, students were not able to attend the laboratory tests. This experience also provided students with an opportunity to communicate not only with each other but also with people in other occupations and to speak with patients as part of a cross-departmental team. Communication is a critical foundational component of The Assembly Education and is an essential theme when considering the continuity of those classes from under-class to upper-class. To measure the effectiveness of IPE, previous quantitative studies have employed the Readiness for Interprofessional Learning Scale (RIPLS),^14^ which is a self-evaluation by students of their learning preparedness. Qualitative research is a method for scientifically examining phenomena that cannot be measured quantitatively.^15,16^ Statistical analysis can reveal behavior patterns but cannot necessarily explain the reason for those patterns. The meaning of behavior patterns and individual experiences can only be understood qualitatively.^17^ Thus, qualitative research is more suitable for obtaining insight into the meaning of communication-related learning for participants compared with quantitative research. In the current study, we therefore used a qualitative research approach to gain an understanding of the Assembly IV trial, particularly the meanings of the lessons that students who participated in IPE in a medical setting learned about communication. Rather than qualitatively evaluating the program, our aim was to understand the learning outcomes related to communication experienced by students who took part in it and to explain that process in as much detail as possible by holding focus groups with participants and conducting interpretive data analysis.

## Methods

This study examined latent meanings rather than attempting to obtain factual information, using an interpretive paradigm.^15,18^

### Study participants

Of the two groups that had undertaken clinical training in the wards of the university hospital, we selected seven candidate participants from Group 1. Students in Group 1 were from all faculties and had taken part in training in the nephrology ward. The research director explained the study to each student individually, then obtained written informed consent.

### Data collection

Focus groups were used to collect data. In a focus group, people with shared experiences are brought together for an open discussion. Interaction with other participants encourages individuals to discuss experiences they have not previously verbalized.^19^

A private room was prepared within the researcher’s facility to maintain participants’ privacy, and a single focus group lasted approximately 1 hour. The audio of the discussions was recorded after obtaining permission from all participants, and a verbatim transcript was created.

The initial focus group was held on December 201x under the guidance of the researchers. The participants were asked, “What did you learn about communication?” and “What sort of changes did you observe in yourself?” The participants discussed communication with patients and communication with other professions in no particular order. To organize and understand both of these areas, an additional focus group was conducted on July 201x+1, asking participants “What did you learn about communicating with patients?” and “What did you learn about communicating with people in other professions?”

### Data analysis

Using the transcript created from the audio recording as data, an analysis was performed using the Steps for Coding and Theorization (SCAT), a method developed by Otani for analyzing qualitative data.^15,20^ The SCAT is an effective method for analyzing small sets of data and can be easily employed by novices. This measure involves listing the segmented data within a matrix, then performing four coding steps in order: <1> identification of words deserving of focus within the data, <2> identification of words external to the text that can be used to restate the focus words, <3> identification of concepts external to the text that explain the focus words, and <4> a coding process using the themes and structural ideas that have arisen.^15^ All of the ideas generated in <4> are then used to create a storyline from which theoretical descriptions can be derived. This entire process was conducted by the author and three individuals who assisted with the research (M.O., K.G., and T.H.) and also served as interviewers.

### Ethical considerations

This study was approved by the medical research ethics review committee of Fujita Health University (approval no. HM x-376). We explained the purpose, methods, and content of the study to participants in an interview. We also explained the qualitative research methods used in the study and informed participants that the data would be disclosed in a way that protected their personal information. We then received written consent from participants. With the disclosure, contact information was provided to each participant so that any individual who decided to withdraw from the study could contact the study representative via email. Moreover, we emphasized that participation in the research was entirely voluntarily, that there were no disadvantages whatsoever for refusing to participate, and that it was possible for participants to withdraw at any time, even after agreeing to take part in the research.

## Results

Of the seven candidate participants, six provided written consent and became the final study participants. Their demographic characteristics are listed in Table 2.

Two main themes arose regarding the communication-related learning of students who participated in the Assembly IV trial. First, the transition from a competing (exclusive) mode to a mutual-understanding mode was revealed by discussions about communication between different professions. Second, the interactive (dialectic) link between involved communication and uninvolved communication was revealed by discussions about communication with patients.

A portion of the SCAT is presented in Table 3 and Table 4, while the derived theoretical descriptions are listed in Table 5 and Table 6. Hereafter, double quotation marks “ ” indicate extracted text data, whereas brackets [ ] indicate structural ideas.

### 1. Transition from a competing (exclusive) mode to a mutual-understanding mode

**Figure 1:**
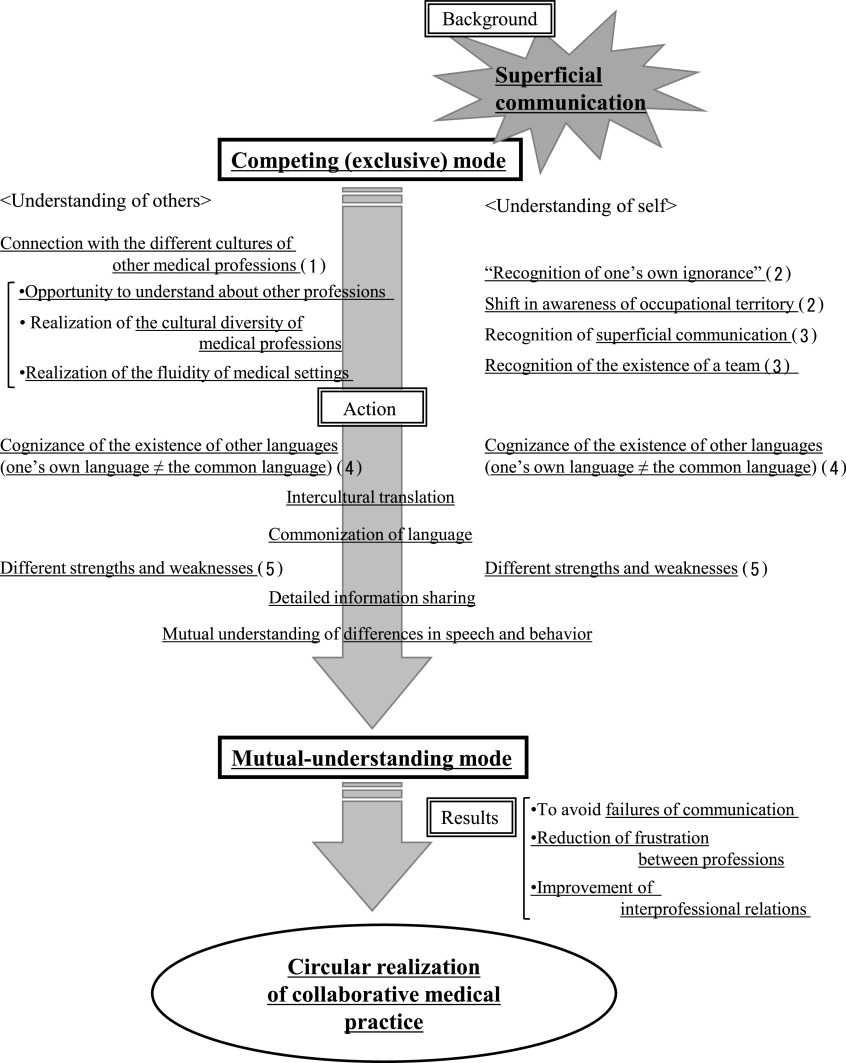
Process of transition from a competing (exclusive) mode to a mutual-understanding mode * In the resulting figure, the structural ideas are underlined.

In communication between different professions, [superficial communication] leads to [a competing (exclusive) mode (i.e., patterns of speech and conduct that occur when an individual attempts to compete with students from other faculties)] and invites failure of the communication process. The transition from [a competing (exclusive) mode] to [a mutual-understanding mode (i.e., patterns of speech and conduct that occur when an individual attempts to reach a mutual understanding with students from other faculties)] takes place through a five-stage process (Figure 1). (1) [Connection with the different cultures of other medical professions] in other faculties with students and people from other occupations in the Assembly IV trial creates [an opportunity to understand about other professions] to recognize features such as [the cultural diversity of medical professions] and [the fluidity of medical settings]. (2) By knowing others, students’ can develop [recognition of one’s own ignorance], with [a shift in awareness of occupational territory]. (3) Recognition of one’s own [superficial communication] through interactions with others and [self-reflection] and leads to [recognition of the existence of a team]. (4) [Cognizance of the existence of other languages (i.e., terminology used in other occupations that cannot be understood in one’s own occupation)] eliminates [the mistaken preconception] that [one’s own language (i.e., terminology understood and used in one’s own occupation)=the common language (i.e., terminology that one’s own occupation and other occupation can understood)]. Then, based on the new awareness that [one’s own language ≠ the common language], [commonization of language] is arrived at via [intercultural translation (i.e., changing terminology that is only used in one occupation to language that can be understood by other occupations)] achieved through [the bracketing of one’s own language (it should be noted that, in phenomenology, this notion refers to suspension of judgment, whereas in the current study it means intentionally suspending the use of language that is exclusively used by one’s own profession)] to facilitate communication with the other party. (5) Finally, [a mutual understanding] of [differences in speech and behavior] as well as [the detailed information sharing] that is inevitably required by things such as [the different strengths and weaknesses] of each occupation and [the realization of the fluidity of medical settings] bring about [transition from a competing (exclusive) mode to a mutual-understanding mode]. This prevents [failure of communication] and helps with [the reduction of frustration between professions] and [improvement of interprofessional relations], achieving [circular realization of collaborative medical practice (i.e., recognizing diverse possibilities and incorporating them into one’s own profession via exposure to the perspectives of people from other occupations and avoiding decision-making based on assumptions that are only valid within one’s own profession; this process enables the discovery of better methods and perspectives and the achievement of effective medical practice)].

### 2. Interactive (dialectic) link between involved and uninvolved communication

Communication with patients can consist of [involved communication] and [uninvolved communication].

[Involved communication (i.e., communication that attempts to connect directly with patients)], which is primarily used by students majoring in nursing or physical therapy, allows for [patient-centric information gathering] through [intimate communication] based on [verbal and nonverbal adroitness]. This ability further develops into [communication with people that extends into their backgrounds (i.e., a perception that includes the personality, social role, and family members of a patient)]. Conversely, students in faculties such as medical technology or radiological technology primarily use [uninvolved communication (i.e., communicating with patients indirectly through data and other methods)], which limits them to [communication with the thing in front of them] because of [a focus on processing specimens] or [a focus on reading images] commonly coupled with [verbal and nonverbal clumsiness].

[Connection with the different cultures of other medical professions] provides an opportunity to recognize the merits of mutual communication. One student in the faculty of medical technology stated: “When I look at a patient ... I connect the test results to that patient.” The students comprehended that a higher-order understanding of people could be attained by making the connection between [an attitude that warmly perceives the feelings of people as individuals] and [an attitude that calmly perceives people as things]. This is [the interactive (dialectic) link between people and things].

One radiological technology student reported seeing the potential in [the coexistence of results (i.e., objective test data) and understanding (i.e., subjective acknowledgment of the patient)] made possible by [the interactive (dialectic) link between people and things], saying that “the end result might be the same (an identical obtained image), but the way that explanations are given may somewhat improve the mood of the patient.”

In addition, [superficial communication] also exists when communicating with patients. Students reported that [a lack of an opportunity to understand about patients] during training within their faculty creates [a hesitation to talk with patients], and therefore they end up [leaving situations to be handled by people in other professions] because of their [awareness of occupational territory].

## Discussion

Students in the Assembly IV trial experienced the process of [transition from a competing (exclusive) mode to a mutual-understanding mode] when communicating with people in other occupations. When carrying out the roles of their individual occupations within a team, students encountered several barriers, such as differences in values, pride, and territorial awareness, and sometimes ended up competing with people in other professions.^21^ However, they reported subsequently achieving a mutual understanding of one another, recognizing the importance of working together to accomplish shared goals, and attempting to modify their behavior. This process can be explained as the transition from the forming stage to the storming stage and then to the norming stage in Tuckman’s team development model.^22^ In addition, considering the Assembly IV trial in terms of the Developmental Model of Intercultural Sensitivity,^23,24^ medical professions could be seen as an aggregation of varying cultural backgrounds. [A competing (exclusive) mode] that occurs with interprofessional communication created a polarized us versus them mentality, arising from [the student’s own pride]. This may reflect a defense against difference so that students could maintain their sense of superiority compared with students in other faculties by demonstrating their own fields of expertise. The students then focused on their commonalities with other students in the same groups or school years as they attempted to perceive others as being at the same level as themselves, with mutual minimization of differences. This can be interpreted as the acceptance of difference in cultural backgrounds and the transition to [a mutual-understanding mode].

Exploring the causes of [a competing (exclusive) mode] can reveal the existence of [superficial communication] between professions. This was seen in the focus group, reflected in the following comments: “I was able to converse normally with my fellow students and instructors in Assembly I through III,” and therefore “I should also be able to attempt communication with other medical professionals.” Students reported that their experiences of speaking with instructors and other students had given them the mistaken impression that their communication skills were sufficient. They had also mistakenly believed that the specialist terminology used in their own professions would be easily understood by people in other professions, but when they attempted to communicate with students in other faculties, they failed because “even within the same medical profession ... we could somehow communicate but not entirely understand each other.” In addition, [superficial communication] was reinforced by [a hesitation to keep asking repeated questions of people in other professions] because of [the student’s own pride]. Students were able to accept this [superficial communication] with people in other professions in the process of defense against difference, minimization of differences, and acceptance of difference against other cultures.^23,24^

The results revealed that [superficial communication] also exists when communicating with patients. This was demonstrated by the following comments: “I completed my studies within my faculty, so I should be able to communicate with patients”; “I had already completed my departmental training ... but when I came in contact with patients this time, I felt the deficiencies in my communication skills”; “When face to face with a patient, I wondered what I should say ...”; “I was scared that I would hurt the patient with my words.” Students realized that they were unable to communicate with patients and that they had previously engaged in [superficial communication].

**Figure 2:**
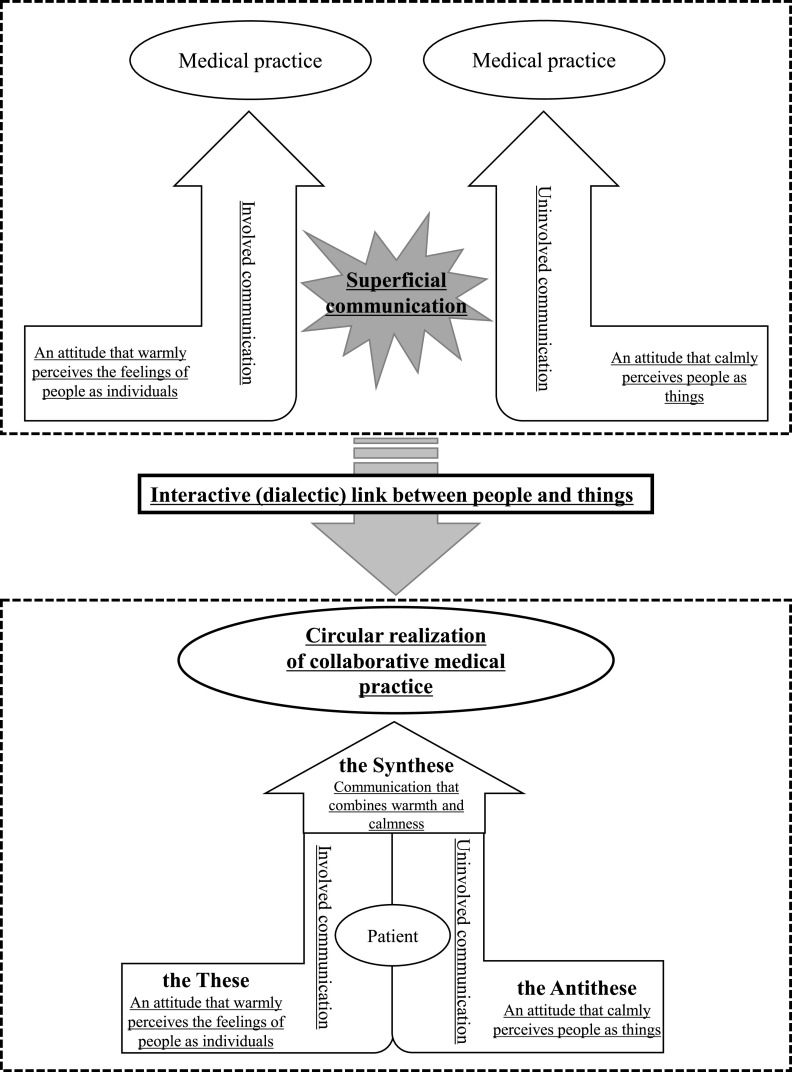
The interactive (dialectic) link between people and things * In this figure, the structural ideas are underlined, and words other than structural ideas are also included.

Patient communication can be categorized into two components: [involved communication] and [uninvolved communication]. The difference between the two stems from variation in professional values, job characteristics, and relationships with patients during treatment. Importantly, these differences are not a measure of correctness or quality. The nurse and the physical therapist focused on personal assistance. Because they spend a lot of time with patients, they placed a high professional value on understanding patients’ lifestyles, emotional states, families, and other background information. As a result, they tended to use [involved communication]. Meanwhile, professionals such as medical technologists and radiological technologists focused on the provision of testing techniques. In accord with their professional roles, they placed a high professional value on [the precision of tests] and the accuracy of their results and appeared to spend a relatively short amount of time with patients. Therefore, they tended to use [uninvolved communication] with patients because their interest lay with specimens, data, and test results. *Dialectics* is a term used in philosophy to refer to how we think about things. From the perspective of dialectics, new ground can be broken by uniting the opposing aspects or contradictions present in things. If we interpret the relationship between [involved communication] and [uninvolved communication] using a dialectical framework, [an attitude that warmly perceives the feelings of people as individuals] (the Thesis) and [an attitude that calmly perceives people as things] (the Antithesis) can be merged to achieve [communication that combines warmth and calmness] (the Synthesis). This enables [the coexistence of results and understanding] regarding patients and [a circular realization of a collaborative medical practice] (Figure 2). Achieving this goal is not about changing the communication style of one’s profession but, rather, incorporating the viewpoint of an opposing communication style into it, providing a dialectic link to create an opportunity to [the avoidance of superficial communication] when interacting with patients.

Radiological technology students also kept in mind the expectation of [the coexistence of results and understanding] of patients gained in the Assembly IV trial, and, when later working as radiological technologists, maintaining an awareness of the lifestyles and backgrounds of the patients they imaged. In other words, students modified their behavior to [future success through patient-centered trial and error learning after graduation], aiming to realize their expectations and implement them in practical medical settings.

As shown in the results, students in the Assembly IV trial (IPE in medical settings) experienced a process of [transition from a competing (exclusive) mode to a mutual-understanding mode] when communicating with people from other professions and acquired the viewpoint of a dialectic link regarding patient communication. This enabled them to [the avoidance of superficial communication] as they deepened their connections with people in other professions, complemented each other’s strengths, and learned about the possibilities inherent in the provision of collaborative medical practice.

### Implications

The findings obtained in this study can provide useful insights regarding facilitators’ preliminary knowledge and knowledge for informing program redesign to effectively implement and improve this program and similar IPE programs at other universities. For example, facilitators may be able to gain a deeper understanding of students’ awareness and the changes resulting from participating in the program. On this basis, facilitators may be better able to select facilities for the program, configure student groups, support students by facilitation during program implementation, and design future programs.

### Limitations

The current findings were based on a relatively small focus group of students in a single year. Therefore, we believe that it is necessary to continue this qualitative research in the future and extend the current findings to enable a more comprehensive and structural understanding of this topic.

## Conclusions

Students who participated in IPE within medical settings learned about the potential to achieve circular realization of collaborative medical practice by moving beyond superficial communication.

## Supplementary Material

Moving beyond superficial communication to collaborative communication: learning processes and outcomes of interprofessional education in actual medical settings

## Figures and Tables

**Table1:** Participants in the Assembly IV trial

Group	Student	Faculty	Place of clinical training
Group 1	Student A	Medicine	University hospital ward (nephrology) Number of beds: 49
	Student B	Nursing	
	Student C	Medical technology	
	Student D	Radiological technology	
	Student E	Rehabilitation (physical therapy)	
	Student F	Clinical engineering	
	Student G	Medical management and information science	
Group 2	Student H	Nursing	University hospital ward (emergency and critical medicine) Number of beds: 43
	Student I	Medical technology	
	Student J	Radiological technology	
	Student K	Rehabilitation (occupational therapy)	
	Student L	Clinical engineering	
	Student M	Medical management and information science	
Group 3	Student N	Medicine	Nursing home Number of beds: 120
	Student O	Nursing	
	Student P	Medical technology	
	Student Q	Radiological technology	
	Student R	Rehabilitation (occupational therapy)	
	Student S	Clinical engineering	
	Student T	Medical management and information science	

**Table2:** Study participants

Student	Faculty	Gender	Age (years)
Student A	Medicine	Female	28
Student B	Nursing	Female	22
Student C	Medical technology	Female	22
Student D	Radiological technology	Male	22
Student E	Rehabilitation (physical therapy)	Female	22
Student G	Medical management and information science	Male	21

**Table3:** Storyline of interprofessional communication in SCAT

A medical student spoke about the importance of the “recognition of one’s own ignorance.” The student realized the fluidity of medical settings. To avoid failures of communication, it is important to transition from a competing (exclusive) mode to a mutual-understanding mode through a mutual understanding of differences in speech and behavior. For example, when there is frustration between professions, the detailed information sharing based on the realization of the fluidity of medical settings can enable the reduction of frustration between professions. This process helps improvement of interprofessional relations. Students’ “recognition of one’s own ignorance” enabled a shift in awareness of occupational territory. The student developed recognition of the existence of a team via a shift in awareness of occupational territory.
A student in the medical technology faculty also spoke about the importance of “recognition of one’s own ignorance.” The Assembly IV trial was also an opportunity to understand about other professions. A mutual understanding of differences in speech and behavior is essential. Through connection with the different cultures of other medical professions, students realized the cultural diversity of medical professions and recognized the existence of superficial communication in their own occupations. Recognizing the isolation of one’s own occupation (i.e., being consumed by their own work) caused students to reevaluate a lack of awareness of patient stemming from a complete focus on the precision of tests and helped them understand the importance of patient awareness. A medical technology student recognized their mindset as a “tester” within the increasing compartmentalization of medical professions. That attitude reinforced the awareness of occupational territory caused by a lack of an opportunity to understand about other professions in their clinical training within their own faculty. The student’s own pride and the mistaken preconception that one’s own language=the common language caused a hesitation to keep asking repeated questions of people in other professions. Cognizance of the existence of other languages caused students to realize that one’s own language ≠ the common language. While Japan’s high-context culture encourages superficial communication, the avoidance of superficial communication using commonization of language will lead to a shared understanding.
A radiological technology student came to a realization about the different cultures of other medical professions, coming to understand that one’s own language ≠ the common language. Commonization of language is critical for intercultural translation achieved through the bracketing of one’s own language. This is important for transition from a competing (exclusive) mode to a mutual-understanding mode based on the understanding of the different strengths and weaknesses.
A nursing student expressed awareness of occupational territory. In addition, in the Assembly IV trial, the student developed awareness of the continuity of patient’ lives from a close-up view, helping them shift from a focus on patients as points to a focus on patients as lines. The student realized the lack of a patient-centric perspective in other professions and aimed for circular realization of collaborative medical practice through patient-centric handover. Commonization of techniques promotes interaction with other professions. Direct common information to complement indirect common information is important.
The Assembly IV trial was an opportunity for connection with the different cultures of other medical professions. It provided an opportunity to understand about other professions, to realize the cultural diversity of medical professions, and to understand the fluidity of medical settings.

**Table4:** Storyline of patient communication in SCAT

A medical student expressed awareness of the continuity of patients’ lives from a birds-eye perspective and recognized the multiple facets of patients. Compared with their previous studies and training, the Assembly IV trial helped the student develop their perspective from curiosity to understanding. Reasons for this transition include differences in prior knowledge, objectives, and member composition.
A medical technology student realized that they had a lack of an opportunity to understand about patients compared with students in other faculties and identified the possibility of changing their self-perception as <testers> with a focus on processing specimens and a tendency to have an insufficient awareness of the individual aspects of patients. The student spoke about the importance of the avoidance of superficial communication through self-reflection about one’s own superficial communication and leaving situations to be handled by people in other professions. A hesitation to talk with patients led to students lagging behind those in other professions. The student recognized that their a lack of awareness of patient stemmed from a complete focus on the precision of tests. The medical technology student recognized the features of nurses that allowed for patient-centric information gathering through intimate communication based on verbal and nonverbal adroitness. The student realized that medical technology students often face challenges such as uninvolved communication and verbal and nonverbal clumsiness. The interactive (dialectic) link between people and things could aid the recognition of other professions, a continuous patient-centric approach, and circular realization of collaborative medical practice.
Like the medical technology student, the radiological technology student recognized that they had a lack of an opportunity to understand about patients and a focus on reading images. The student expressed that their own clinical training limited their communication with the thing in front of them by promoting uninvolved communication. However, the Assembly IV trial enabled them to develop the skills for communication with people that extends into their backgrounds of patients via involved communication. The radiological technology student realized the importance of shifting their focus from “things” to “people”. The student realized that there was an attitude that warmly perceives the feelings of people as individuals and an attitude that calmly perceives people as things. The student arrived at an expectation that the interactive (dialectic) link between people and things could enable the coexistence of results and understanding regarding patients and could lead to future success through patient-centered trial and error learning after graduation.
A nursing student expressed awareness of the continuity of patients’ lives. The student expected communication that combines warmth and calmness would enable circular realization of collaborative medical practice. Nursing training is designed to focus on the execution of tasks, and thus the curriculum within the nursing department had a rigid framework. However, the Assembly class was more flexible, broadening the perspectives of students by allowing them to engage in critical thinking in a way that they were not able to under the standard nursing model. Please note that the pronoun “they” is used elsewhere to refer to this student. Please choose one pronoun to use consistently to refer to this student.

**Table5:** Theoretical descriptions of interprofessional communication in SCAT

•	In hospital wards, there is frustration between professions.
•	Medical professions involve the different cultures of other medical professions, the cultural diversity of medical professions, the isolation of one’s own occupation, and increasing compartmentalization of medical professions.
•	Superficial communication enhances a competing (exclusive) mode.
•	There is awareness of occupational territory.
•	A shift in awareness of occupational territory can enable recognition of the existence of a team.
•	“Recognition of one’s own ignorance” is important.
•	Students’ “recognition of one’s own ignorance” enabled a shift in awareness of occupational territory.
•	Differences in speech and behavior require a mutual understanding.
•	A mutual understanding of differences in speech and behavior prompts transition from a competing (exclusive) mode to a mutual-understanding mode.
•	Transition from a competing (exclusive) mode to a mutual-understanding mode avoid failures of communication.
•	There is a transitional process from a competing (exclusive) mode to a mutual-understanding mode.
•	Transition from a competing (exclusive) mode to a mutual-understanding mode can prevent failure of communication.
•	The detailed information sharing based on the realization of the fluidity of medical settings can enable the reduction of frustration between professions.
•	The detailed information sharing leads to an improvement of interprofessional relations.
•	Connection with the different cultures of other medical professions provides an opportunity to understand about other professions, to realize the cultural diversity of medical professions, and to realize the fluidity of medical settings.
•	Realizing the different cultures of other medical professions allows one to recognize superficial communication in one’s own profession.
•	A lack of an opportunity to understand about other professions reinforces awareness of occupational territory.
•	There is the mistaken preconception that one’s own language=the common language.
•	The mistaken preconception and the student’s own pride can cause a hesitation to keep asking repeated questions of people in other professions.
•	Cognizance of the existence of other languages allows one to realize that one’s own language ≠ the common language.
•	Japan’s high-context culture encourages superficial communication.
•	Commonization of language allows one to avoid superficial communication.
•	The avoidance of superficial communication enables a shared understanding.
•	Commonization of language is important for intercultural translation via the bracketing of one’s own language.
•	It is important to transition from a competing (exclusive) mode to a mutual-understanding mode based on an understanding of the different strengths and weaknesses.
•	Transition from a competing (exclusive) mode to a mutual-understanding mode enables circular realization of collaborative medical practice. Please check that this is your intended meaning.

**Table6:** Theoretical descriptions of patient communication in SCAT

•	There is a hesitation to talk with patients.
•	A hesitation to talk with patients leads students lagging behind those in other professions.
•	Superficial communication stems from being content to leaving situations to be handled by people in other professions.
•	Assembly IV provides students with the opportunity for self-reflection about their own superficial communication.
•	Self-reflection enables students to recognize the importance of the avoidance of superficial communication.
•	Nurses use their skills in verbal and nonverbal adroitness to engage in intimate communication based on involved communication.
•	Nurses allow for patient-centric information gathering through intimate communication.
•	Intimate communication allows for patient-centric information gathering.
•	Involved communication develops into communication with people that extends into their backgrounds.
•	The medical technology student used uninvolved communication due to verbal and nonverbal clumsiness.
•	Uninvolved communication by students limits communication with the thing in front of them.
•	A complete focus on the precision of tests leads to a lack of awareness of patient.
•	The interactive (dialectic) link between people and things enables recognition of other professions, a continuous patient-centric approach, and circular realization of collaborative medical practice.
•	The interactive (dialectic) link between people and things enables the coexistence of results and understanding regarding patients.
•	Students put their expectation of the coexistence of results and understanding into practice with future success through patient-centered trial and error learning after graduation.
•	The medical technology and radiological technology students felt that they are a lack of an opportunity to understand about patients.
•	The medical technology student had a focus on processing specimens.
•	The radiological technology student had a focus on reading images.
•	There is an attitude that warmly perceives the feelings of people as individuals and an attitude that calmly perceives people as things.
•	Communication that combines warmth and calmness could enable circular realization of collaborative medical practice.
