# Supplementary material for: Moving beyond superficial communication to collaborative communication: learning processes and outcomes of interprofessional education in actual medical settings
Source: Fujita Med J. 2020 Jul 14;6(4):93–101. doi: 10.20407/fmj.2019-026 (PMC8761828; doi:10.20407/fmj.2019-026)
Supplement: Supplementary file 1 — Moving beyond superficial communication to collaborative communication: learning processes and outcomes of interprofessional education in actual medical settings [file fmj-6-093-s001.pdf]

原著論文

見せかけのコミュニケーションから協働的コミュニケーションへの移行
−医療現場における専門職連携教育の学修過程とそのアウトカム−

Moving beyond superficial communication to collaborative communication : learning processes and outcomes of interprofessional education in actual medical settings

伊藤美保子[1]，　肥田武[2]，　後藤和恵[3]，　後藤道子[4]，　金田嘉清 [1]，　大槻眞嗣,[3,5]

キーワード: 医療現場、専門職連携教育、SCAT、解釈的分析、コミュニケーション

要旨

目的：本研究では、プログラムに参加した学生のフォーカスグループとその解釈的な分析によって、プログラムの中で学生に生じた学修成果とその過程を可能な限り詳細かつ深く解明することを目的とした。

方法：病棟における多学科混成の臨床実習に参加した学生を研究参加者として質的研究を行った。この研究では解釈的パラダイムに依拠して、事象の事実情報の立証よりも潜在的意味の検討を重視した。また、フォーカスグループによりデータを採取し、Steps for Coding and Theorization (SCAT) を用いて分析した。

結果：アセンブリ IV トライアル（医療現場における専門職連携教育）に参加した学生は、職種間コミュニケーションにおける張り合い（排他的）モードから分かり合いモードへの移行プロセスを経験し、対患者コミュニケーションにおける関与のコミュニケーション（注釈：直接、患者に関わろうとする姿勢でのコミュニケーション）と非関与のコミュニケーション（注釈：直接ではなく、データ等を介した患者とのコミュニケーション）の対話的（弁証法的）接続という視点を獲得した。これらは、見せかけのコミュニケーションからの脱却により、他職種との関わりを深め、互いの特長を補い、理想の医療を提供することに繋がる可能性があることを学んだ。

結論：医療現場における専門職連携教育に参加した学生は、見せかけのコミュニケーションから脱却することが、“理想の医療”の協働による円環的実現、即ち、自職種だけの思い込みに囚われて判断せず、他職種の視点にもさらされることにより、多様な可能性が提案され、自職種の元へ戻るプロセスを経て良い捉え方や方法が見つかり、理想的な医療が実現することに繋がる可能性があることを学んだ。

1　藤田医科大学保健衛生学部リハビリテーション学科
2　一宮研伸大学看護学部看護学科
3　藤田医科大学医学部医学教育企画室
4　三重大学大学院医学系研究科生命医科学専攻臨床医学系講座家庭医療学分野
5　藤田医科大学医学部医学科
470-1192, 愛知県豊明市沓掛町田楽ケ窪 1-98（e-mail: mohtsuki@fujita-hu.ac.jp）

緒言

現在、わが国では、医療の高度化、分業化が急速に進み、医療の安全性と質保障に対する国民からの要求水準が次第に高くなっている。また、社会の高齢化に即して、地域社会における高齢者の複雑な問題も増加し、専門職連携実践（interprofessional work：IPW）が必須となっている。それゆえ、準備教育として卒前に専門職連携教育[1]（interprofessional education：IPE）を実践する必要がある。専門職連携教育と専門職連携実践の枠組み[2]が、世界保健機関（World Health Organization：WHO）により発表され専門職連携教育が世界的に推奨されている。わが国における最近の調査において、保健医療福祉分野で IPE を実施している学校、学科は増加傾向にある[3]。教育技法としては講義、教室での小グループ学習が多く[3,4]、医療現場における IPE は少ない[5,6]。学修成果基盤型教育（Outcome-based education：OBE）[7]では、卒業時の learning outcome が提示され[8,9,10]、IPE の学修成果（learning outcome）も報告されている[5,11]。わが国における IPE の枠組み[12]のコア・ドメインは「Patient-/Client-/Family-/Community-centered」と「Interprofessional Communication」である。

藤田医科大学では、1964 年の建学時からチーム医療の基盤作りのために、１、2 年生の全学生を対象とした多学科合同の授業としてアセンブリ教育を行ってきた。１年生のアセンブリ教育（アセンブリⅠ）と２年生のアセンブリ教育（アセンブリⅡ）では、他者とのコミュニケーションを促しつつ、多彩なプロジェクト学修を選択制で提供した。その後、2013 年に３年生以上を対象とした IPE としてのチーム基盤型学修（Team-based learning：TBL）を導入し、高学年におけるアセンブリ教育を開始した[13]。主に３年生を対象としたアセンブリ教育（アセンブリⅢ）では、患者を中心に考える視点を提示し、模擬事例によるディスカッションを求めた。臨床実習、又は臨地実習を経験していない３年生までのアセンブリ教育は、学生間、学生─教員間のコミュニケーションを経験する場となったが、対患者および専門職間のコミュニケーションを学ぶ機会ではなかった。そこで、201x 年に医療現場における各学科での実習を終えた４年生以上を対象とした選択制プログラムとしてのアセンブリⅣトライアルを実施した。

201x 年９月 11 日から９月 15 日に、本学医学部 4 年生 2 名、医療科学部６学科４年生 18 名の合計 20 名が参加し、３つの多学科混成チームに分かれ、大学病院の病棟（腎臓内科、救急総合内科）と老人保健施設でそれぞれ実習を行った（表 1）。学生が他職種の役割を理解した上で、自職種と他職種の連携を学ぶことを学修目標とした。グループ１の学生は大学病院の腎臓内科病棟で、グループ２の学生は大学病院の救急総合内科病棟で実習を行った。グループ１とグループ２の学生は、担当患者の診療情報をカルテと病棟スタッフより収集し、担当患者の病室を訪れた。また、担当患者が受ける検査（エックス線撮影、心電図検査）に病棟外へも同行した。学生は、患者の病状変化に対応する病棟の医療従事者の動きを観察したり、退院調整のための多職種参加型のカンファレンスにも参加した。一方、グループ３の学生は、民間の老人保健施設で実習を行った。認知症に罹患した利用者を担当し、施設における利用者の生活を見学し、認知症状のある利用者と会話をしながら情報収集を行うなどした。施設には臨床検査技師や診療放射線技師は常駐しておらず、検査に立ち会うことはなかった。アセンブリ Ⅳトライアルは、学生にとって、学生間ではあるが職種間コミュニケーションを実施し、多学科混成チームで対患者コミュニケーションを経験する学修機会となった。とりわけ、コミュニケーションについては、アセンブリ教育を支える根幹的な要素であり、アセンブリ教育の低学年から高学年への連続性を考える上でも重要なテーマである。

IPE の教育効果を測る場合、学生の学修準備性の自己評価である the Readiness for Interprofessional Learning Scale（RIPLS）[14]などを用いた量的研究が報告されている。一方、量的に測れないものを科学的に考える方法として、質的研究がある[15,16]。統計分析では、行動パターンは示せるが、その理由は説明できない。人々の行動パターンや経験にどんな意味があるのかは、質的にしか解明できない[17]。コミュニケーションに関する学びの意味についての知見を得るには、量的研究よりも、質的研究の方が適している。そこで、

本研究では、アセンブリⅣトライアル、即ち、医療現場における専門職連携教育に参加した学生のコミュニケーションについての学びの意味を知るために、質的研究を行った。尚、我々は、『プログラムの質的な評価』を目指したのではなく、プログラムに参加した学生のフォーカスグループとその解釈的な分析によって、プログラムの中で学生に生じた学修成果とその過程を可能な限り詳細かつ深く解明することを目的とした。

方法

この研究は、解釈的パラダイムに依拠して、事象の事実情報の立証よりも潜在的意味の検討を重視するものである[15,18]。

研究参加者

大学病院の病棟における臨床実習をした２グループのうち、全学科の学生が参加した腎臓内科病棟で実習を行った１グループ７名を研究参加候補者とした。研究責任者から個別に本研究の主旨を説明し、書面による同意を得た。

データ採取

データ採取には、フォーカスグループを用いた。フォーカスグループは、同様な体験を共有する人々に集まってもらい、同時に話を聞くことで、参加者間の相互作用によって、個人が言語化していなかった体験の言語化を促進する[19]。

場所は、研究責任者の所属機関における研究参加者のプライバシーが保てる個室を準備し、１回１時間程度とした。会話内容は研究参加者の同意を得て録音し、逐語記録を起こした。

第１著者による進行の下で、201x 年 12 月に初回フォーカスグループを行った。「コミュニケーションに関して、何を学んだか」、「自分自身にどのような変化がみられたか」を質問項目として設定した。研究参加者がコミュニケーションについて、対患者コミュニ

表１　アセンブリ Ⅳ トライアル参加学生

グループ	学生	学科	実習場所
グループ１	学生 A	医学科	大学病院（腎臓内科病棟）
	学生 B	看護学科	49 病床
	学生 C	臨床検査学科	
	学生 D	放射線学科	
	学生 E	リハビリテーション学科（理学療法専攻）	
	学生 F	臨床工学科	
	学生 G	医療経営情報学科	
グループ２	学生 H	看護学科	大学病院（救急総合内科病棟）
	学生 I	臨床検査学科	43 病床
	学生 J	放射線学科	
	学生 K	リハビリテーション学科（作業療法専攻）	
	学生 L	臨床工学科	
	学生 M	医療経営情報学科	
グループ３	学生 N	医学科	老人保健施設
	学生 O	看護学科	入所：120 床
	学生 P	臨床検査学科	通所：30 名
	学生 Q	放射線学科	
	学生 R	リハビリテーション学科（作業療法専攻）	
	学生 S	臨床工学科	
	学生 T	医療経営情報学科	

ケーションと職種間コミュニケーションの両者を混在したまま語った。そこで、両者を整理して把握するため、201x+1 年 7 月に追加のフォーカスグループを行い、「対患者コミュニケーションに関して、何を学んだか」、「職種間コミュニケーションに関して、何を学んだか」を尋ねた。

データ分析

音声記録から作成した逐語録をデータとし、大谷が開発した質的データ分析手法である Steps for Coding and Theorization（SCAT）[15,20] を用いて分析した。SCAT は、初学者が容易に着手でき、小規模データの分析にも有効な方法である。SCAT は、マトリックスの中にセグメント化したデータを記載し、〈１〉データ中の注目すべき語句、〈２〉それを言いかえるためのテクスト外の語句、〈３〉それを説明するようなテクスト外の概念、〈４〉そこから浮き上がるテーマ・構成概念の順に４ステップのコーディング手続きを踏む[15]。その後、〈４〉の概念を全て用いてストーリー・ラインを作成し、理論記述を導く。この全過程をインタビュアーでもある研究分担者３名（M.O，K.G，T.H）と第１著者で行った。

倫理的配慮

この研究は、藤田医科大学医学研究倫理委員会（承認番号：HMx-376）によって承認された。我々は研究参加者に研究の目的、方法、そして、フォーカスグループを含む内容を説明した。我々は、研究参加者に質的研究が行われること、個人情報が守られてどのように情報が発表されるかについて、書面による説明と同意を得ることについても説明した。発表に際しては、研究参加者が同意を撤回することが出来るよう連絡先に関する情報も提供した。さらに、我々はこの研究が自由意志によって任意で実施されたこと、即ち、拒否しても何等不利益を被ることのないこと、そして、研究参加者はたとえ、研究に協力することに同意した後でも、いつでも同意を撤回することが出来ることを説明した。

結果

研究参加候補者７名の内、６名から書面による同意を取得し、研究参加者とした。研究参加者の属性を表２に示す。

アセンブリⅣトライアルに参加した学生のコミュニケーションについての学びとして、特徴的な２つの主題が浮かび上がった。１つは職種間コミュニケーションについての語りから得られた、張り合い（排他的）モードから分かり合いモードへの移行であった。もう１つは対患者コミュニケーションについての語りから得られた、関与のコミュニケーションと非関与のコミュニケーションの対話的（弁証法的）接続であった。

SCAT による分析の一部（ストーリー・ライン）を表３と表４に、導き出された理論記述を表５と表６に示した。なお、本文中の「」は抽出したテキストデータ、【】は構成概念を意味する。

１．張り合い（排他的）モードから分かり合いモードへの移行

職種間コミュニケーションにおいて【見せかけのコミュニケーション】が【張り合い（排他的）モード[註1]】を導き、コミュニケー

表２　研究参加者

学生	学科	性別	年齢
学生 A	医学科	女	28
学生 B	看護学科	女	22
学生 C	臨床検査学科	女	22
学生 D	放射線学科	男	22
学生 E	リハビリテーション学科（理学療法専攻）	女	22
学生 G	医療経営情報学科	男	21

表３　職種間コミュニケーションについてのストーリー・ライン

医学生は、”知らなさを知る”ことの重要性を語った。医学生は医療現場の流動性に気づいた。コミュニケーションの不成立を避けるためには、言動の違いの相互理解による張り合い（排他的）モードから分かり合いモードへの移行が重要である。例えば、職種間フラストレーションがおきるときには、医療現場の流動性への気づきを前提とした逐一の情報共有が職種間フラストレーションの軽減をもたらす。これは職種間関係の向上につながる。”知らなさを知る”ことで、職種テリトリー意識の変化が生じた。また、職種テリトリー意識の変化からチームの存在への気づきも得られた。

検査学科学生も、”知らなさを知る”の重要性を語った。アセンブリⅣトライアルは、他職種を知る機会でもある。言動の違いの相互理解が必要である。医療職内異文化との関わりによって医療職の多文化性に気づき、自職種の見せかけのコミュニケーションを認識した。自職種の孤島性（自職種に関する業務へのこだわり）への気づきは、検査精度への徹底的なこだわりゆえの対患者意識の希薄化を自己反省させ、対患者意識の必要性を実感させた。医療職の「たこつぼ化」の中で、検査学科学生は「検査の人」の姿への気づきを得た。それは自科実習における他職種を知る機会の不足によって職種テリトリー意識が強化された姿である。自言語＝共通語という誤った先入観とプライドが他職種への聞き返しへのためらいをもたらす。それに対して異言語（職種特有の言葉）の存在の認知が自言語≠共通語に気づかせる。察しの日本文化は見せかけのコミュニケーションを促進するが、言語の共通化による見せかけのコミュニケーションからの脱却が共通理解を成立させる。

放射線学科学生も、自言語≠共通語を認識した。これについては自言語の括弧入れを経た異文化間翻訳による言語の共通化が必要である。得手不得手の相違を前提とした、張り合い（排他的）モードから分かり合いモードへの移行が重要である。

看護学生も職種テリトリー意識を持っていた。しかし、アセンブリⅣでは虫瞰的視点による患者生活の連続性への気づきを得て、点の患者中心性から線の患者中心性へと移行した。他職種における患者中心性の不足に気づき、患者中心性のリレーによって“理想の医療”の協働による円環的実現につながるのではと訴えた。技術の共通化によって他職種との互助的関与が促進される。間接的情報共有を補う直接的情報共有が必要である。

アセンブリⅣトライアルでの医療職内異文化との関わりは、他職種を知る機会であり、医療職の多文化性に気づく機会であり、医療現場の流動性に気づく機会であった。

表４　対患者コミュニケーションについてのストーリー・ライン

医学生は、鳥瞰的視点による患者生活の連続性への気づきを得て、患者の顔の多重性の認識に至った。早期体験実習と比べてアセンブリⅣでは、物珍しさから理解への昇華が生じた。その要因として、既修得知識の違い、目的の違い、メンバー構成の違いを挙げている。

検査学科学生は、他職種に比べて患者を知る機会の不足に気づき、検体処理中心で患者の顔の個別性の不認識状態にある「検査の人」の姿の変容の可能性を指摘した。患者への話しかけの躊躇いが他職種への出遅れを招き、他職種任せに甘んじていた見せかけのコミュニケーションへの自己反省により、見せかけのコミュニケーションからの脱却の必要性を語った。検査精度への徹底的なこだわりゆえの対患者意識の希薄化に気づいた。検査学科学生は、看護師の特長を言語や非言語の巧みさや関与のコミュニケーションに基づき、親密なコミュニケーションによる患者中心の情報取得を可能にすることだと捉えた。それに対して、検査技師の課題は、言語や非言語の拙さや非関与のコミュニケーションであると捉えた。新たな「検査の人」の姿の実現のための「物」から「者」へのシフトの必要性に気付いた。つまり者と物との対話的（弁証法的）接続により他職種からの承認を得られ、患者中心性のリレーや“理想の医療”の協働による円環的実現を可能にするということである。

放射線学科学生は、検査学科学生同様に患者を知る機会の不足と、読影中心であることに気づいた。また、自科実習＝「点」の実習では非関与のコミュニケーションによる目の前の物とのコミュニケーションに止まっていた。しかし、アセンブリ実習＝「線」の実習を通して関与のコミュニケーションによる背景まで広がる者とのコミュニケーションへと発展した。自科実習は、自職種アイデンティティ獲得のための土壌づくり、アセンブリ実習は、患者中心性促進のための肥料まきと位置付けた。放射線学生は、「物」から「者」へのシフトの必要性に気付いた。人を者として感情を伴って温かく捉える態度と人を者として冷静に捉える態度があることに気づいた。者と物との対話的（弁証法的）接続が患者にとっての結果と納得の両立を招くと期待するようになり、卒後の患者中心的試行錯誤への結実に至っている。

看護学生は、患者生活の連続性への気づきを得た。彼女は温かさと冷静さを合わせたコミュニケーションが、“理想の医療”の協働による円環的実現を可能にすることに期待した。看護実習は課題遂行への張り付きが多く、自科実習＝厳格な枠づけであったのに対し、アセンブリ実習＝緩やかな枠づけであり、それにより看護の規範にとらわれない思索を行い、見解の広がりを得た。

表5　職種間コミュニケーションについての理論記述

- 病棟では、職種間フラストレーションがおきる。
- 医療職には、医療職内異文化、医療職の多文化性、自職種の孤島性、医療職のたこつぼ化が存在する。
- 見せかけのコミュニケーションが張り合い（排他的）モードを強化する。
- 職種テリトリー意識がある。
- 職種テリトリー意識の変化からチームの存在への気づきを得る。
- “知らなさを知る”ことは重要である。
- ”知らなさを知る”ことで、職種テリトリー意識の変化が生じる。
- 言動の違いの相互理解が必要である。
- 言動の違いの相互理解により張り合い（排他的）モードから分かり合いモードへの移行が起きる。
- 張り合い（排他的）モードから分かり合いモードへの移行プロセスがある。
- 張り合い（排他的）モードから分かり合いモードへの移行によりコミュニケーションの不成立が回避できる。
- 医療現場の流動性への気づきを前提とした逐一の情報共有が職種間フラストレーションの軽減をもたらす。
- 逐一の情報共有は、職種間関係の向上になる。
- 医療職内異文化との関わりは、他職種を知る機会であり、医療職の多文化性に気づく機会であり、医療現場の流動性に気づく機会となる。
- 医療職の多文化性への気づきは、自職種の見せかけのコミュニケーションを認識させる。
- 他職種を知る機会の不足により職種テリトリー意識が強化される。
- 自言語＝共通語という誤った先入観がある。
- 誤った先入観とプライドが他職種への聞き返しへのためらいをもたらす。
- 異言語の存在の認知が自言語≠共通語に気づかせる。
- 察しの日本文化は見せかけのコミュニケーションを促進する。
- 言語の共通化が見せかけのコミュニケーションから脱却させる。
- 見せかけのコミュニケーションからの脱却が共通理解を成立させる。
- 自言語の括弧入れを経た異文化間翻訳による言語の共通化が必要である。
- 得手不得手の相違を前提とした、張り合い（排他的）モードから分かり合いモードへの移行が重要である。
- 張り合い（排他的）モードから分かり合いモードへの移行により“理想の医療”の協働による円環的実現に至る。

表6　対患者コミュニケーションについての理論記述

- 患者への話しかけの躊躇いがある。
- 患者への話しかけの躊躇いが他職種への出遅れを招く。
- 他職種任せに甘んじていた見せかけのコミュニケーションがある。
- アセンブリⅣは、見せかけのコミュニケーションを自己反省させる。
- 自己反省により、見せかけのコミュニケーションからの脱却の必要性を認識させる。
- 看護師は、言語や非言語の巧みさにより関与のコミュニケーションに基づく親密なコミュニケーションを実践する。
- 看護師は、親密なコミュニケーションによる患者中心の情報取得を行う。
- 親密なコミュニケーションは患者中心の情報取得を可能にする。
- 関与のコミュニケーションは、背景まで広がる者とのコミュニケーションへ発展する。
- 検査技師は、言語や非言語が拙く、非関与のコミュニケーションである。
- 非関与のコミュニケーションは、目の前の物とのコミュニケーションに止まる。
- 検査精度への徹底的なこだわり故の対患者意識の希薄化を生む。
- 者と物との対話的（弁証法的）接続は、他職種からの承認、患者中心性のリレー、“理想の医療”の円環的実現を可能とする。
- 者と物との対話的（弁証法的）接続が患者にとっての結果と納得の両立を招く。
- 結果と納得の両立への期待が、卒後の患者中心的試行錯誤への結実に至る。
- 検査学科の学生と放射線学科の学生は、患者を知る機会が不足している。
- 検査学科の学生は検体処理中心である。
- 放射線学科の学生は、読影中心である。
- 人を者として感情を伴って温かく捉える態度と人を物として冷静に捉える態度がある。
- 温かさと冷静さを合わせたコミュニケーションは者と物との対話的（弁証法的）接続である。

ションの不成立を招く。【張り合い（排他的）モード】から【分かり合いモード[註2]】へ移行するには、５段階のプロセスを経る（図１）。①アセンブリⅣトライアルでの他学科学生や他職種という【医療職内異文化との関わり】が、【医療職の多文化性】や【医療現場の流動性】に気づくための【他職種を知る機会】となる。②他者を知ることで自分の【“知らなさを知る”[註3]】ことができ、【職種テリトリー意識の変化】が生じる。③他者とのやりとりや【自己省察】を通して自分の【見せかけのコミュニケーション】を認識し、【チームの存在への気づき】が導かれる。④医療職内の【異言語[註4]の存在の認知】により、【自言語[註5]＝共通語[註6]】という【誤った先入観】を払拭し、【自言語≠共通語】とうい新たな認識のもと、相手に通じさせるために【自言語の括弧入れ[註7]】を経た【異文化間翻訳[註8]】による【言語の共通化】を行う。⑤各職種の【得手不得手の相違】や【医療現場の流動性への気づき】から必然的に求められる【逐一の情報共有】と、【言動の違い】の【相互理解】が、【張り合い（排他的）モードから分かり合いモードへの移行】をもたらす。これにより【コミュニケーションの不成立】が回避され、【職種間フラストレーションの軽減】と【職種間関係の向上】に繋がり、【“理想の医療”の協働による円環的実現[註9]】となる。

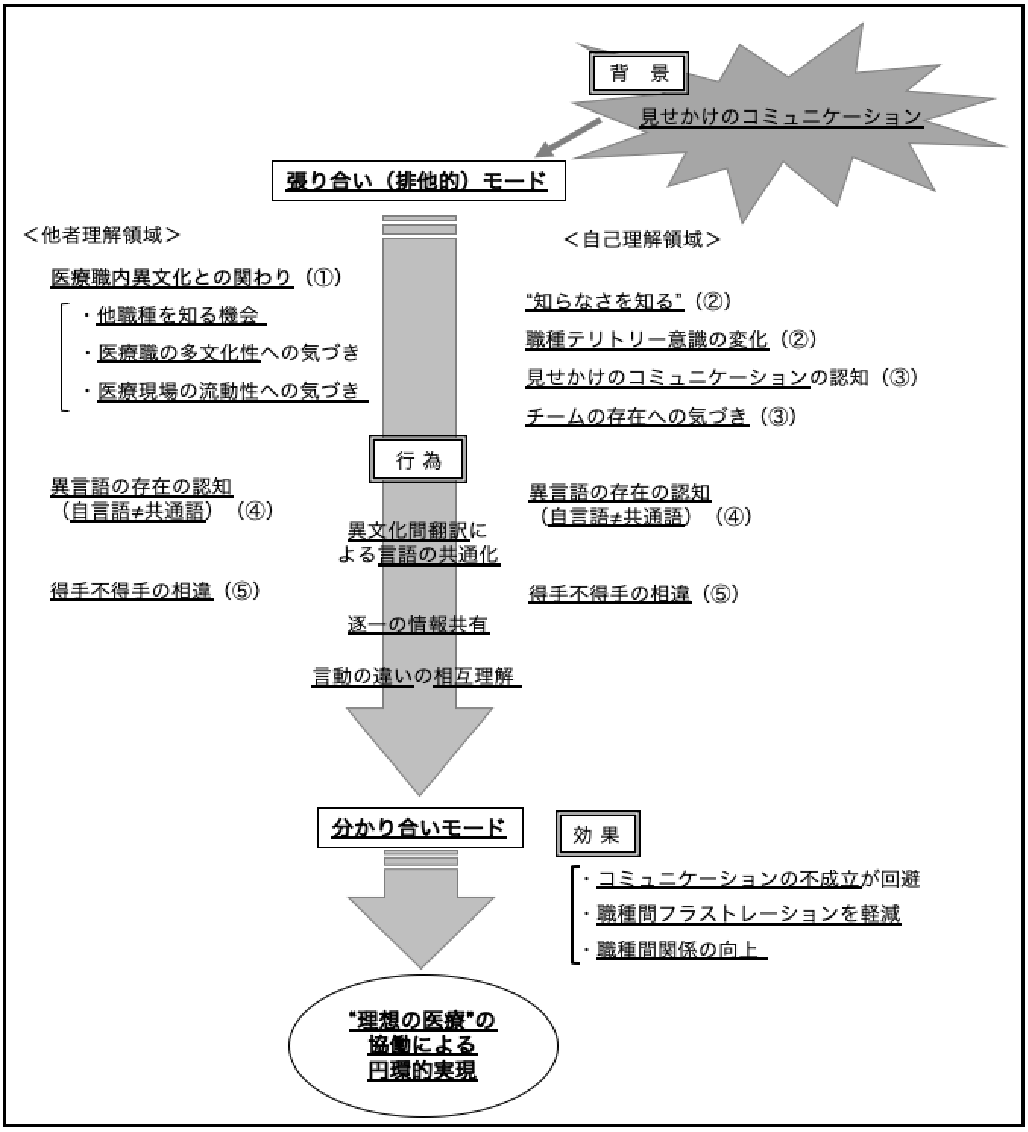

図１　張り合い（排他的）モードから分かり合いモードへの移行プロセス

＊下線を付した語句は SCAT を用いた分析における＜４＞の概念である。

２．関与のコミュニケーションと非関与のコミュニケーションの対話的（弁証法的）接続

対患者コミュニケーションにおいて、【関与のコミュニケーション】と【非関与のコミュニケーション】が存在する。

主に看護学科の学生やリハビリテーション学科（理学療法専攻）の学生がとる【関与のコミュニケーション[註10]】は、【言語や非言語の巧みさ】に基づき、【親密なコミュニケーション】による【患者中心の情報取得】を可能とする。これはさらに、患者の【背景まで広がる者[註11]とのコミュニケーション】へ発展する。一方、主に臨床検査学科の学生や放射線学科の学生がとる【非関与のコミュニケーション[註12]】は、目の前の【検体処理中心】や【読影中心】の規範に【言語や非言語の拙さ】も相まって【目の前の物とのコミュニケーション】にとどまる。

【医療職内異文化との関わり】は、互いのコミュニケーションの特長に気づく機会ともなる。臨床検査学科の学生の「（中略）患者さんを見て（中略）、検査の値とその患者さんを結び付けられる」というテクストがある。すなわち学生は、【人を者として感情を伴って温かく捉える態度】と【人を物として冷静に捉える態度】とを結びつけることによって、より高次元の人間理解に到達できることを理解した。これは、【者と物との対話的（弁証法的）接続】である。

放射線学科の学生は【者と物との対話的（弁証法的）接続】によって、「結果は同じ（得られる画像は同じ）かもしれないが、説明の仕方で患者さんの気持ち、説明された側の気持ちはちょっとよくなる（のではないか）」という患者にとっての【結果[註13]と納得[註14]の両立】が得られることへの期待を抱いた。

また、対患者コミュニケーションにおいても【見せかけのコミュニケーション】が存在する。自科実習での【患者を知る機会の不足】により、【患者への話しかけの躊躇い】が生じ【職種テリトリー意識】を理由に【他職種任せ】となる。

考察

アセンブリ IV トライアルにおいて、学生は職種間コミュニケーションにおける【張り合い（排他的）モードから分かり合いモードへの移行】というプロセスを経験した。学生はチームにおいて自職種としての役割を果たす時、価値観の違い、プライド、テリトリー意識といった障壁[21]に阻まれ、他職種と張り合った。その後、お互いを理解して共有された目標を達成するために協働することの大切さに気づき、行動変容を試みた。このプロセスは Tuckman's team development model における形成期（forming stage）から混乱期（storming stage）を経て、統一期（norming stage）に移行する過程にあたると解釈されうる[22]。また、アセンブリIVトライアルを異文化感受性発達モデル[23,24]の枠組みで考えると、医療における専門職は、異なった文化的背景を持つ集合体であると捉えることができる。職種間コミュニケーションにおける【張り合い（排他的）モード】では、自分の【プライド】に固執して“自分 vs 彼ら”という二極化を招いた。これは自分の得意分野を示すことで他学科学生に対する優位性を保つための『違いからの防衛』であった。その後、同じグループ、同じ学年といった共通部分に注目し、相手を同質化し、お互いの『違いの最小化』を試みた。これはお互いの文化的背景の『違いの受容』であり、【分かり合いモード】へ移行したのだと解釈することができる。

【張り合い（排他的）モード】を生じた原因を探索すると、職種間における【見せかけのコミュニケーション】の存在が浮かび上がった。これは、「アセンブリ I から III で学生同士や先生とは普通に話せてきた」のだから「医療職同士のコミュニケーションも（普通に）図れるだろ」などのテクストから抽出された。学生は他の学生や教員と会話した経験により、自分達に充分なコミュニケーション能力があると誤解していた。また、学生は自職種の用いる専門用語は他職種に容易に通じると考え、他学科学生とのコミュニケーションを試みたが、「同じ医療職ではあるけれども（中略）会話になっていそうでなってない」といった状況に陥った。また、【プライド】から生じる【他職種への聞き返しへの躊躇い】も【見せかけのコミュニケーション】を強化させる一因となった。他職種に対する【見せかけのコミュニケーション】は、異文化に対する『違いからの防衛』、『違いの最小化』、『違いの受容』の過程[23,24]の中で脱却していくことができたと考える。

【見せかけのコミュニケーション】は、対患者コミュニケーションにおいても存在した。これは、学生の「自科実習も終えているから患者ともコミュニケーションが取れるだろう」、「すでに学科の実習をさせてもらったけど（中略）、今回参加して自分が患者さんと接する時のコミュニケーション能力の低さを実感した」、「患者さんをいざ前にした時にどうやって話していいのだろう（中略）」、「自分の発言が患者を傷つけてしまわないだろうか」などのテクストから抽出された。自分が患者に対してうまくコミュニケーションをとることができず、【見せかけのコミュニケーション】をとっていたことに気づいた。

対患者コミュニケーションには【関与のコミュニケーション】と【非関与のコミュニケーション】の２つの特長があった。両者の違いは、専門職の持つ価値観や職務特性、医療行為における患者との関係性などの相違であり、善悪や優劣を測れるものではない。看護師や理学療法士は対人援助中心の職種であり、患者と接する時間が長く、患者の生活や心情、家族などの背景にも配慮することに職業特性や職業的価値を持つ。それゆえに【関与のコミュニケーション】をとる傾向がある。一方、臨床検査技師や診療放射線技師は患者と関わる時間が短く、検査技術の提供が中心の職種であり、検査精度や結果の正確性に職業特性や職業的価値を持つ。それゆえ、検体やデータ、検査結果に関心を寄せるので、患者に対して【非関与のコミュニケーション】をとる傾向がある。ものの考え方を表わす哲学の用語のひとつに弁証法がある。弁証法では、ものの対立や矛盾の検討を通して、その昇華的な統一により一層高い境地に進むという見方をする。【関与のコミュニケーション】と【非関与のコミュニケーション】の関係を弁証法の枠組みで解釈すると、【人を者として感情を伴って温かく捉える態度】『正（These）』と、【人を物として冷静に捉える態度】『反（Antithese）』とを結びつけ、【温かさと冷静さを合わせたコミュニケーション】『合（Synthese）』に至る。これにより、患者にとっての【結果と納得の両立】や【“理想の医療”の協働による円環的実現】がもたらされる（図 2）。自職種のコミュニケーションのスタイルを変えるのではなく、その視点に相反するコミュニケーションスタイルの視点を取り入れること、つまり弁証法的接続により、対患者に対する【見せかけのコミュニケーションからの脱却】の一機会を得たと考えられる。また、放射線学科の学生は、アセンブリ IV トライアルで抱いた、患者にとっての【結果と納得の両立】への期待を、診療放射線技師として撮影時に患者の背景や生活場面を意識して対応することを心がけた。つまり放射線学科の学生は【卒後の患者中心的試行錯誤への結実[註15]】という行動変容を起こした。

以上のように､医療現場における専門職連携教育としてのアセンブリⅣトライアルにおいて､学生は職種間コミュニケーションにおける【張り合い（排他的）モードから分かり合いモードへの移行】のプロセスを経験し､対患者コミュニケーションにおける弁証法的接続という視点を獲得した。これらにより【見せかけのコミュニケーションからの脱却】を可能とし､他職種との関わりを深め､互いの特長を相補し､理想の医療を提供することに繋がる可能性があることを学んだ。

今後の展望

この研究で得られた知見は、アセンブリ教育および他大学の同様の IPE プログラムの有効な実施や改善のため、ファシリテータの事前知識やプログラムの再設計の側面で有益な示唆を得られる可能性がある。つまり、このプログラムを指導する教員（ファシリテータ）が、このプログラムの参加により生じる学生の気づきや変化について、事前により深い理解を得られる可能性がある。それに基づいて、プログラム実施施設の選択、学生グループの構成、プログラム実施中のファシリテータから学生への学修支援、そして将来のプログラムの再設計がより適切に行えるようになる可能性がある。

本研究の限界

ただし、今回の知見は、限られた年度の限られた学生のフォーカスグループに基づくものであり、今後さらに同様の研究を継続して、今回の知見を、より包括的で構造的なものへと発展させていく必要があると考えている。

学んだ。

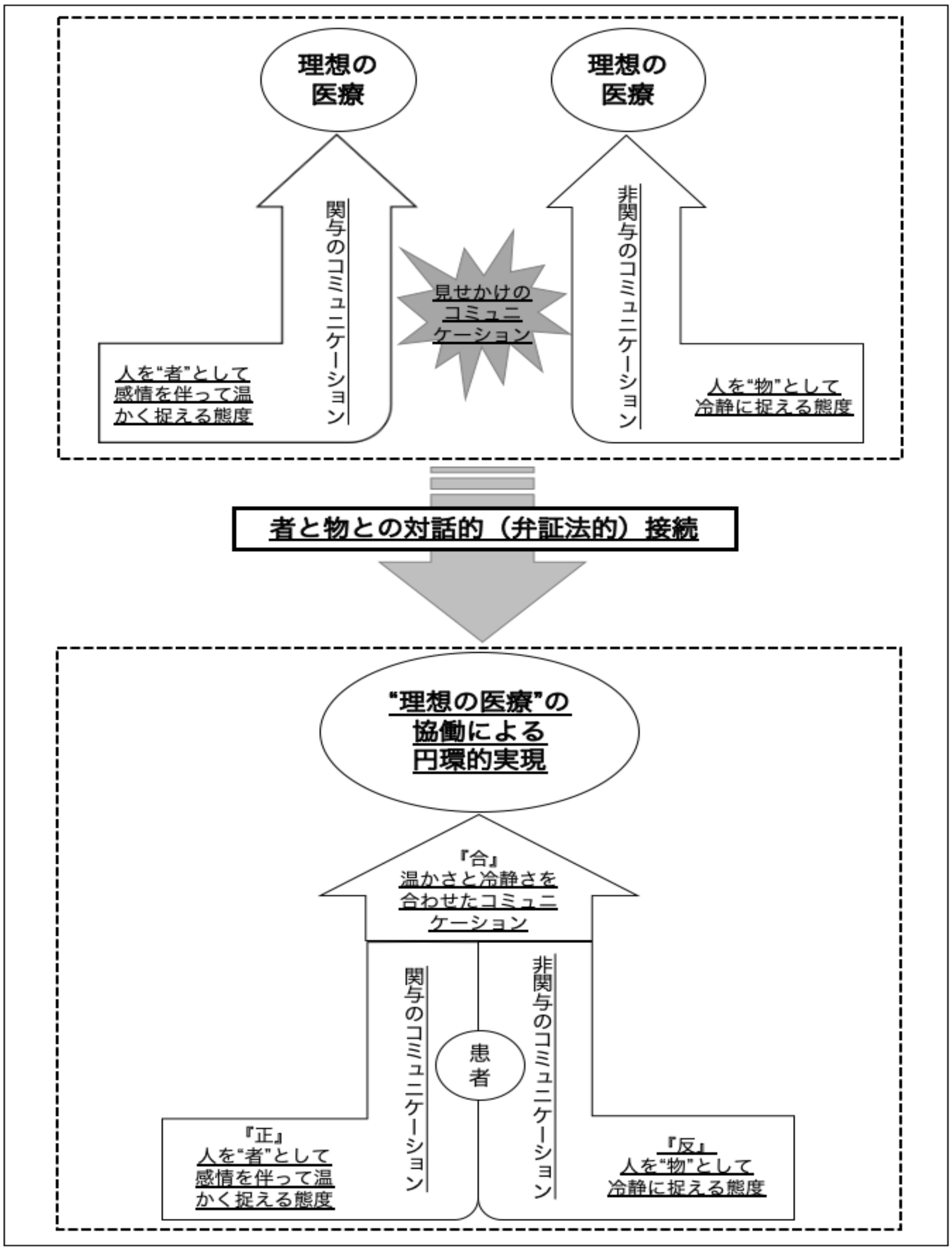

図２ 者と物との対話的（弁証法的）接続

＊下線を付した語句は SCAT を用いた分析における＜４＞の概念である。

結論

医療現場における専門職連携教育に参加した学生は、見せかけのコミュニケーションから脱却することで、“理想の医療”の協働による円環的実現に繋がる可能性があることを学んだ。

利益相反

この研究に関して報告する利益相反はない。

謝辞

私たちの研究に参加してくれたすべての研究参加者と、私たちの研究をサポートしてくださった井上敬子さんに感謝致します。

引用文献

1. The Centre for the Advancement of Interprofessional Education. Interprofessional education today, yesterday and tomorrow. <https://www.caipe.org/resources/publications/caipe-publications/caipe-2002-interprofessional-education-today-yesterday-tomorrow-barr-h> (Accessed June 30, 2019).
2. World Health Organization. Framework for action on interprofessional education and collaborative practice. <https://www.who.int/hrh/resources/framework_action/en/> (Accessed June 30, 2019).
3. Ogawa S, Takahashi Y, Miyazaki M. The current status and problems with the implementation of interprofessional education in Japan. Journal of Research in Interprofessional Practice and Education 2015; 5: 1-15.
4. Goto M, Haruta J, Oishi A, Yoshida K, Yoshimi K, Takemura Y, Yoshimoto H. A cross-sectional survey of interprofessional education across 13 healthcare professions in Japan. The Asia Pacific Scholar 2018; 3: 38-46.
5. Thistlethwaite J, Moran M. Learning outcomes for interprofessional education(IPE): Literature review and synthesis. J Interprof Care 2010; 24: 503-13.
6. Reeves S, Pelone F, Harrison R, Goldman J, Zwarenstein M. Interprofessional collaboration to improve professional practice and healthcare outcome. Cochrane Database Syst Rev 2017; 6: CD000072.
7. Harden RM, Crosby JR, Davis MH, Friedman M. AMEE Guide No. 14: Outcome-based education: Part 5-From competency to meta-competency: a model for the specification of learning outcomes. Med Teach 1999; 21: 546-52.
8. General Medical Council. Good medical practice; 2013. <https://www.gmc-uk.org/-/media/documents/good-medical-practice---english-1215_pdf-51527435.pdf> (Accessed June 23, 2019).
9. Englander R, Cameron T, Ballard AJ, Dodge J, Bull J, Aschenbrener CA. Toward a common taxonomy of competency domains for the health professions and competencies for physicians. Acad Med 2013; 88: 1088-94.
10. Frank JR, Snell L, Sherbino J. CanMEDS 2015 Physician Competency Framework; 2015. <http://canmeds.royalcollege.ca/uploads/en/framework/CanMEDS%202015%20Framework_EN_Reduced.pdf > (Accessed June 23, 2019).
11. The Canadian Interprofessional Health Collaborative. Interprofessional Education & Core Competencies LITERATURE REVIEW; 2007. <http://www.cihc.ca/files/publications/CIHC_IPE-LitReview_May07.pdf> （Accessed June10,2019）
12. Haruta J, Yoshida K, Goto M, Yoshimoto H, Ichikawa S, Mori Y, Yoshimi K, Otsuka M. Development of an interprofessional competency framework for collaborative practice in Japan. J Interprof Care 2018; 32: 436-43.
13. Ohtsuki M, Matsui T. Large-scale team-based learning for interprofessional education in medical and health sciences. Med Teach 2014; 36: 452-3.
14. Parsell G, Bligh J. The development of a questionnaire to assess the readiness of health care students for interprofessional learning (RIPLS). Med Educ 1999; 33: 95-100.
15. 大谷尚：質的研究の考え方−研究方法論から SCAT による分析まで−．名古屋大学出版会．愛知．2019；pp.20-116, 30-32, 184-188, 270-368．
16. Maeno T, Takayashiki A, Anme T, Tohno E, Maeno T, Hara A. Japanese students’ perception of their learning from an interprofessional education program: a qualitative study. Int J Med Educ 2013；4: 9-17.
17. Richards L. Handing qualitative data. Los Angeles: Sage; 2014: 8-14.
18. Wilson, TP. Conceptions of Interaction and Forms of Sociological Explanation. American Sociological Review 1970; 35:697-710.
19. 大谷尚：質的研究とは何か．薬学雑誌．2017；pp.137:653-58．
20. 大谷尚：SCAT: Steps for Coding and Theorization−明示的手続きで着手しやすく小規模データに適用可能な質的データ分析手法−．日本感性工学会論文誌．2011；10: pp.155-60．
21. Visser CLF, Ket JCF, Croiset G, Kusurkar RA. Perceptions of residents, medical and nursing students about Interprofessional education: a systematic review of the quantitative and qualitative literature. BMC Med Educ 2017; 17: 77.
22. TuckmanBW. Developmental sequence in small groups. Psychol Bull 1965; 63: 384-39.
23. Bennett MJ. A developmental model of intercultural sensitivity. <https://www.idrinstitute.org/wp-content/uploads/2018/02/FILE_Documento_Bennett_DMIS_12pp_quotes_rev_2011.pdf> (Accessed Jun 6,2019).
24. 山本志都：文化的差異の経験の認知−異文化感受性発達モデルに基づく日本的観点からの記述−．多文化関係学．2014；11: pp.67-86．

註釈

註[1] 張り合い（排他的）モード：他学科の学生と張り合おうとする言動パターン

註[2] 分かり合いモード：他学科の学生と分かり合おうとする言動パターン

註[3] SCAT では名詞または名詞句を構成概念とするがここでは変則的に文を用いたため""を付けた

註[4] 異言語：自職種では理解できない他職種の用語

註[5] 自言語：自職種で理解され使用される用語

註[6] 共通語：注釈：自職種と他職種が理解できる用語

註[7] 自言語の括弧入れ：現象学では判断の停止を意味するが、この研究では自分の職業で使用されている自言語の使用を意図的に停止することを意味する

註[8] 異文化間翻訳：ある職種においてのみ通用する用語を他職種でも理解できる用語にかえること

註[9] “理想の医療”の協働による円環的実現：自職種だけの思い込みに囚われて判断せず、他職種の視点にもさらされることにより、多様な可能性が提案され、自職種の元へ戻る。こうしたプロセスを経て良い捉え方や方法が見つかり、理想的な医療が実現すること

註[10] 関与のコミュニケーション：直接、患者に関わろうとする姿勢でのコミュニケーション

註[11] 背景まで広がる者：患者の性格や社会的役割、家族までを含めて捉える

註[12] 非関与のコミュニケーション：直接ではなく、データ等を介した患者とのコミュニケーション

註[13] 結果：客観的な検査データ

註[14] 納得：主観的な患者の得心

註[15] 卒後の患者中心的試行錯誤への結実：期待の実現を目指して卒後の臨床で実践すること
